# Design and Potential of Non-Integrating Lentiviral Vectors

**DOI:** 10.3390/biomedicines2010014

**Published:** 2014-01-27

**Authors:** Aaron Shaw, Kenneth Cornetta

**Affiliations:** 1 Department of Medical and Molecular Genetics, Indiana University School of Medicine, Indianapolis, IN 46202, USA; E-Mail: aarshaw@iupui.edu; 2 Department of Medicine, Indiana University School of Medicine, Indianapolis, IN 46202, USA; 3 Department of Microbiology and Immunology, Indiana University School of Medicine, Indianapolis, IN 46202, USA

**Keywords:** HIV, non-integrating, episome, lentiviral, integrase

## Abstract

Lentiviral vectors have demonstrated promising results in clinical trials that target cells of the hematopoietic system. For these applications, they are the vectors of choice since they provide stable integration into cells that will undergo extensive expansion *in vivo*. Unfortunately, integration can have unintended consequences including dysregulated cell growth. Therefore, lentiviral vectors that do not integrate are predicted to have a safer profile compared to integrating vectors and should be considered for applications where transient expression is required or for sustained episomal expression such as in quiescent cells. In this review, the system for generating lentiviral vectors will be described and used to illustrate how alterations in the viral integrase or vector Long Terminal Repeats have been used to generate vectors that lack the ability to integrate. In addition to their safety advantages, these non-integrating lentiviral vectors can be used when persistent expression would have adverse consequences. Vectors are currently in development for use in vaccinations, cancer therapy, site-directed gene insertions, gene disruption strategies, and cell reprogramming. Preclinical work will be described that illustrates the potential of this unique vector system in human gene therapy.

## 1. Introduction

Gene therapy using lentiviral vectors (LV) holds great promise for the treatment of a wide variety of disorders. The major advantage of these vectors is their ability to stably integrate into target cells, thus providing genetic modification of the cell and all of its progeny. Other advantages of LVs include low immunogenicity, a lack of prior immunity, a relatively large packaging capacity, and an ability to be pseudotyped with alternative envelopes thus altering vector tropism [1,2,3,4,5,6,7,8]. To date, the predominant use of these vectors has been *ex vivo* modification of hematopoietic stem cells, T cells or other targets where the transduced cell is expected to expand *in vivo*. For example, early clinical trials have shown evidence of disease correction or delay of onset including treatment for thalassemia, adrenoleukodystrophy, chronic lymphocytic leukemia, metachromatic leukodystrophy, and Wiskott Aldrich syndrome [9,10,11,12,13].

A number of potential lentiviral vector applications will not require stable integration, including their use in immunizations, cytotoxic cancer therapies, or delivery to sites such as the central nervous system. In these settings, it may be advantageous to express the vector transgenes episomally to mitigate the risk of insertional mutagenesis. The phenomenon of insertional mutagenesis occurs when regulatory regions in the vector activate surrounding genes involved with cell growth or the integration disrupts genes resulting in growth dysregulation or genetic instability [9,14,15].

In this review, we will reveal how the unique life-cycle of the lentivirus permits the design of non-integrating lentiviral vectors. Specifically, modifying integrase and/or its binding site allows the development of episomally expressed vectors that retain the ability to infect target cells and express the transgene(s) of choice. We also describe the use of non-integrating lentiviral vectors in gene therapy applications, their limitations, and current advances intended to improve upon clinical utility.

### 1.1. Lentiviral Vector Design

**Figure 1:**
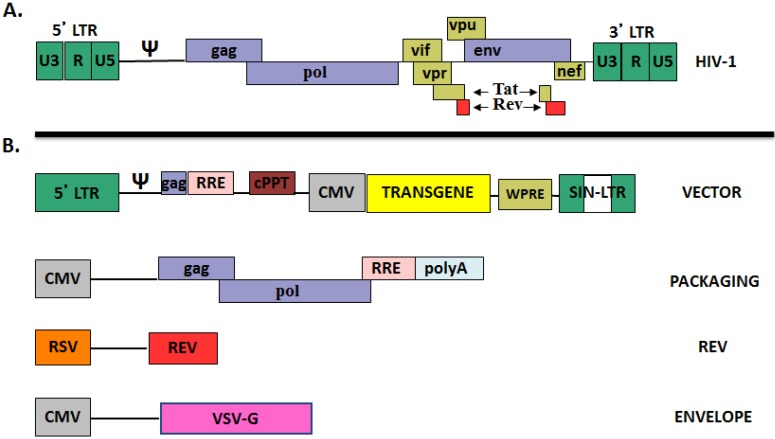
Schematic of HIV-1 and Third Generation Lentiviral Packaging System. (**A**) The HIV-1 Virus contains three gene regions *gag*, *pol*, and *env* along with accessory proteins and the flanking Long Terminal Repeats (LTR); (**B**) The lentiviral components found in the four plasmids used in generating third generation lentiviral vectors. The vector plasmid contains a self-inactivating 3' LTR (SIN-LTR), a Rev responsive element (RRE), a central polypurine tract (cPPT), and the Woodchuck Hepatitis Virus Post-Transcriptional Response Element. The psi sequence (Ψ) allows for efficient incorporation of the vector RNA genome into particles. In this schematic, the CMV early promoter is used for transgene expression but other promoters are commonly substituted. The packaging plasmid expresses the *gag* and *pol* gene regions of HIV-1 which encode proteins required for virion formation and vector processing. This plasmid also contains a RRE. A plasmid expressing *rev* is provided to facilitate nuclear transport of RRE containing transcripts. The fourth plasmid is the envelope plasmid. Lentiviral vectors are commonly pseudotyped with the Vesicular Stomatitis Virus G glycoprotein (VSV-G) as an alternative to the native HIV-1 envelope to increase the range of cell types and animal species susceptible to vector transduction.

Lentiviral vectors derived from the human immunodeficiency virus type 1 (HIV-1) have the ability to integrate efficiently into quiescent or non-dividing cells [16]. As depicted in Figure 1A, LVs were advanced by removing non-essential sequences and genomic regions involved with viral replication and virulence from the wild-type lentiviral genome (e.g., *nef*, *vif*, *vpr*, and *vpu*). The end result is a replication defective vector containing the necessary elements for packaging and processing but still capable of integration [17,18]. Vector particles are generated using a series of plasmids that express the vector genome and the viral proteins required for particle formation. The technology for packaging vector particles continues to evolve, but the commonly used “third generation” systems utilize a series of four vector plasmids that are introduced into cells by transient transfection (Figure 1B) [19]. In addition to the transgene plasmid containing the vector genome, the system uses a plasmid expressing the *gag* and *pol* gene regions that produce the HIV-1 structural proteins required for capsid formation and genome integration. A plasmid expressing HIV-1 *rev* is also included to activate the *rev* responsive element engineered into the transgene and gag/pol plasmids. This facilitates nuclear transport and is also included as a safety feature. The fourth plasmid expresses an envelope glycoprotein that engages receptors on the target cells. As the native HIV-1 glycoprotein is generally restricted to CD4 positive cells, investigators utilize alternative envelopes, most commonly the Vesicular Stomatitis Virus G glycoprotein [20] (VSV-G), to facilitate uptake into a wide variety of species and cell types. The use of multiple plasmids and the requirement for *rev* are included to minimize recombination events that would lead to the development of a replication competent virus.

For efficient integration, viral particles must contain the proteins encoded in the HIV-1 pol region which are necessary for vector processing including reverse transcriptase, polymerase and protease. Cellular proteins important in efficient transduction include cyclophilin A and integrase interactor 1 which are also packaged within the capsid structure [21,22,23,24,25,26,27,28]. Vector particles also contain two copies of a single-stranded RNA genome. As shown in Figure 1A, each end of the RNA genome contains a long-terminal repeat (LTR), with an untranslated 5' and 3' segment (U5/U3) flanking a repeat region (R). Most vectors retain certain components of the HIV-1 genome including the psi signal that markedly increases the packaging of the genome into the mature virion. A short portion of *gag* sequence which is critical for generating high titer vector is also retained.

The transgene plasmid contains the minimal components of HIV-1 required for vector production and integration. The transgene of interest is 3' to a promoter element that regulates expression. The choice of the specific promoter is driven by the intended use. Common examples include tissue specific promoters, enhancers, insulators, or microRNA regions. Additional elements are added to increase vector production and/or expression including a polypurine tract [29,30] and the Woodchuck Hepatitis Virus Post-Transcriptional Response Element (WPRE) [31,32].

An important safety feature of most LVs is the inclusion of a Self-Inactivating Long Terminal Repeat (SIN-LTR). This feature minimizes the risk of producing a replication-competent lentivirus by recombination with wild-type viruses. The mechanism involves taking advantage of the normal replication cycle of HIV-1. In wild-type HIV-1, the viral promoter is within the U3 region of the 5' LTR and is required to generate the full length viral transcript. The U3 region is also present in the 3' LTR but is not essential in the DNA form of the virus. During viral replication, the RNA genome is reverse transcribed and the 3' LTR is utilized in formation of both the 5' and 3' LTR of the daughter virus. By incorporating a large deletion into the U3 region of the 3' LTR any progeny will contain two inactivated LTR after reverse transcription [17,33]. Transgene expression is dependent solely on the internal promoter (for example, the CMV promoter engineered into the vector plasmid as illustrated in Figure 1B).

### 1.2. Retaining Key Parts of the Life Cycle during Non-Integrating Lentiviral Vector Design

**Figure 2:**
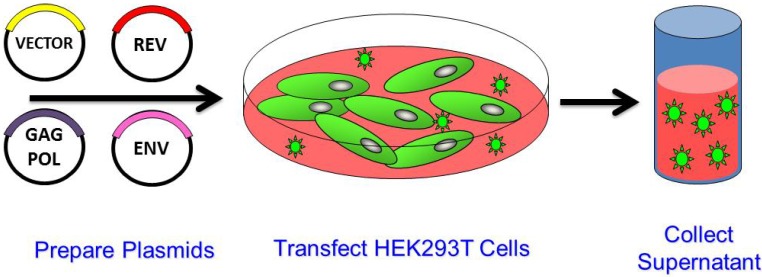
Generation of Lentiviral Vector by Transient Transfection. The four packaging plasmids are transfected into cells that have a high capacity for vector production. The most commonly used cell line is HEK293T. Maximal vector production occurs 48–72 h after transfection. The vector particles are released into the media which is collected and clarified of cell debris. Vector particles can be further purified and/or concentrated.

LVs are generated by introducing the transgene and packaging plasmids into cells, most commonly HEK293T cells (Figure 2). Vector supernatant is collected from the media and typically contains between 10^5^ and 10^7^ infectious units per milliliter. This is dependent on the vector design as the addition or deletion of elements to vector design can affect vector titer. LV can be concentrated by ultracentrifugation and clinical vector products are usually purified using a combination of chromatography, tangential flow filtration and diafiltration [34,35,36].

In order to effectively transduce a target cell, both integrating and non-integrating lentiviral vectors (NILV) must retain the ability to readily enter the cell, form a pre-integration complex, be transported into the nucleus and efficiently express its genetic payload. Depending on the envelope pseudotype used the membrane bound LV particles enter cells either by direct fusion with the plasma membrane [2] or via a receptor-mediated endosomal pathway [20]. In the direct fusion pathway the LV is uncoated upon entry to release the viral contents into the cytoplasm. This allows for reverse transcription of the viral RNA into linear cDNA and development of the pre-integration complex (PIC). The PIC consists of the reverse transcribed viral cDNA complexed with integrase, matrix, reverse transcriptase, and nucleocapsid proteins [37,38,39,40]. The endosomal pathway is dependent upon the pH within the endosome for membrane fusion, subsequent uncoating, and PIC formation within the cytoplasm. The transportation of the PIC to the nucleus is not completely understood, but is believed to occur by an ATP-dependent process [41] via nucleoporins [42,43] using nuclear localization signals and cellular transport mechanisms [44]. Certain of the known localization signals have been removed during LV design; nevertheless, the transduction of quiescent cells by LV is well documented.

Understanding the processing of the LV cDNA after reverse transcription is important when designing NILV. Normally the linear LV cDNA generated during reverse transcription enters the nucleus with the linear 2-LTR form [45] representing the preferred substrate for integration [46,47]. A small portion of LV genomes can persist episomally as linear cDNA, 2-LTR circular forms or 1-LTR circular forms. The majority of 2-LTR circles are formed through non-homologous end-joining of the 5' and 3' LTRs [46,48,49]. The majority of 1-LTR circles are formed by homologous recombination between the LTRs [49,50,51,52,53,54], as an aberrant product of incomplete reverse transcription [55,56,57,58,59], or through alternative mechanisms such as autointegration [60]. It is these episomal forms that allow for the stable expression of the vector transgene without integration in non-dividing cells [61,62,63,64].

## 2. Designing Non-Integrating Lentiviral Vectors

### 2.1. Inhibiting Integration/Developing NILV

When designing NILV it is important that modifications maintain the vectors ability to enter their target cells, perform reverse transcription, transport the PIC into the nucleus and efficiently express their transgene product. This entails selectively inhibiting or altering only the aspects of the vectors lifecycle that lead to integration. As integrase-mediated catalysis is the primary means of integration for LV, the inhibition of its function is necessary in the development of NILV.

**Figure 3:**
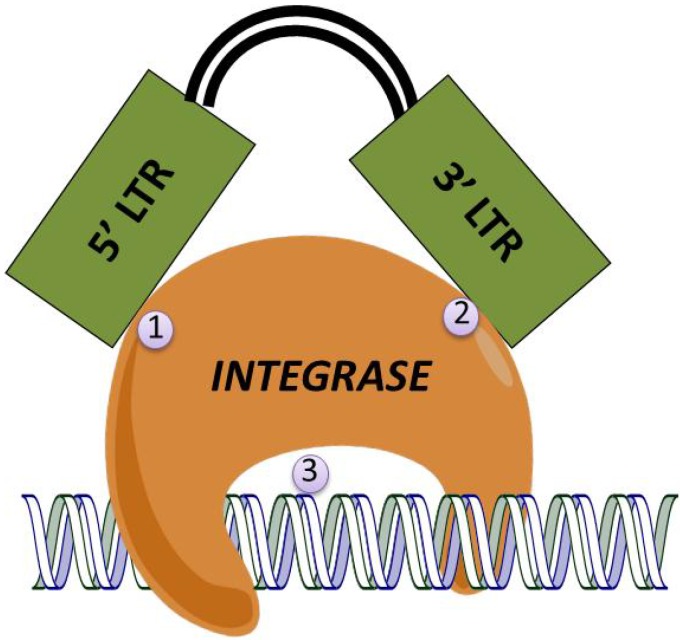
Targets for Creation of Non-Integrating Lentiviral Vectors. Three strategies have been devised to generate non-integrating vectors. Mutations are generated in (**1**) a 12 base-pair region of the U3 region of the 5' LTR; (**2**) an 11 base-pair region of the U5 region of the vector’s 3' LTR; or (**3**) point mutations in the integrase protein that interfere with processing of the vector DNA. This depiction is simplified as integrase and the vector DNA are part of a complex of proteins.

Normally, integration into the target cell’s genome is mediated by the viral integrase. This protein first binds to the viral cDNA at attachment sites located within the U3 region of the 5' LTR and the U5 region of the 3' LTR [65,66,67,68]. Integrase processes the 3' ends of the viral cDNA leaving a CA dinucleotide overhang [69,70] and then attaches the recessed ends to the 5' phosphorylated ends of a double-stranded cut made in the target genome. Integrase then repairs the gaps resulting in a 5 base-pair repeat flanking the inserted vector genome [70,71]. Figure 3 shows the three general points that can be targeted in developing a NILV; mutations in the integrase protein that alter its ability to process the target cell chromosomal DNA or alteration in the vector LTRs that prevent integrase from attaching to the 5' or 3' LTR.

The most common target for inhibiting viral integration is mutation of the integrase protein producing integrase-defective lentiviral vectors. LV integrase is coded for by the HIV-1 Pol region and the region cannot be deleted as it encodes other critical activities including reverse transcription, nuclear import, and viral particle assembly [72,73]. Mutations in *pol* that alter the integrase protein fall into one of two classes: those which selectively affect only integrase activity (Class I); or those that have pleiotropic effects (Class II) [74]. Mutations throughout the *N* and *C* terminals and the catalytic core region of the integrase protein generate Class II mutations that affect multiple functions including particle formation and reverse transcription [74,75,76]. However, class II mutations are not suitable when designing NILVs because they disrupt functions that are critical for vector processing and expression.

Class I mutations limit their affect to the catalytic activities, DNA binding, linear episome processing and multimerization of integrase [77]. The most common Class I mutation sites are a triad of residues at the catalytic core of integrase, including D64, D116, and E152 [78,79]. Each mutation has been shown to efficiently inhibit integration with a frequency of integration up to four logs below that of normal integrating vectors while maintaining transgene expression of the NILV [61,80,81].

Another alternative method for inhibiting integration is mutations in the integrase DNA attachment site (LTR *att* sites) within a 12 base-pair region of the U3 or an 11 base-pair region of the U5 regions at the terminal ends of the 5' and 3' LTRs, respectively [61,68,80,82,83]. These sequences include the conserved terminal CA dinucleotide which is exposed following integrase-mediated end-processing. Single or double mutations at the conserved CA/TG dinucleotide result in up to a three to four log reduction in integration frequency [80]; however, it retains all other necessary functions for efficient viral transduction.

### 2.2. Minimizing Illegitimate Integration

While NILV can significantly reduce the frequency of integration there still remains a low level of vector integration [61,62,80,81,84,85,86,87,88,89]. Integration associated with NILV has been studied using insertion site analysis and high-throughput sequencing. These analyses indicate that the integration observed is not integrase-mediated as the insertion sites lack the canonical features of LTR end-processing including the five base-pair repeat of genomic DNA flanking at the site of vector insertion [80,85,88,90,91]. The vector inserts also vary with some containing fully intact sequence, truncations at the terminal ends of the LTRs, or insertions/deletions of genomic DNA flanking the vector. A significant number of these illegitimate integrations are occurring at sites of chromosomal breakage and are mediated by non-homologous end-joining mechanisms [90,92]. It may be possible to impede illegitimate integration of NILV by inhibition of cellular factors in the double-strand break (DSB) repair pathway. In a recent study by Koyama and colleagues, introduction of an ataxia telangiectasia mutated specific inhibitor (KU55933) consistently blocked DSB-specific integration in wild-type and integrase deficient LV [90]. Whether or not inhibitors to DNA repair can be used clinically to limit non-integrase mediated integration remains to be determined.

Another method for further reducing the frequency of illegitimate integration is limiting the linear form of the vector DNA. Linear DNA has been shown to integrate much more efficiently than supercoiled DNA [46,47]. The linear form also appears to be the preferred substrate for both integrase and non-integrase-mediated insertion. One approach has been to limit linear 2-LTR episomal forms by inducing the formation of circular episomal forms. For example, Kantor and colleagues have shown that deleting the vectors 3' polypurine tract (PPT) results in aberrant reverse transcription leading to the preferential formation of 1-LTR circular episomes and a reduction in linear forms [58]. Using this strategy they were able to reduce the frequency of integration by 10-fold when using an integrating vector. Of relevance to NILV, this modification reduced the frequency of integration of an integrase deficient LV by another 3-fold over integrase deficient LV without the modification. As newer modifications are developed to reduce integration they will need to be tested experimentally to ensure there is no reduction in the level of transgene expression.

It should be noted that LTR *att* site mutations have been directly compared to point mutations in integrase. The consensus is that mutations to integrase provide a greater reduction in the frequency of integration. Yanez-Munoz *et al.* estimated the frequency of reversion mutations in NILV to reach 1/815 [89]. The point mutations to LTR *att* sites could carry a higher rate of reversions but whether LTR *att* site mutations with larger deletions will reduce the frequency of illegitimate integration remains to be determined. Interestingly, while mutations to integrase and the LTR att sites independently inhibit integration efficiently, some studies suggest there are no synergistic effects upon combining these mutations to further reduce integration by a vector [61,80].

### 2.3. Optimization of NILV Expression

NILV are associated with a significantly reduced level of transgene expression as compared to a normal integrating LV [16,58,61,62,64,84,87,93,94,95]. This remains a key issue in developing clinically effective NILV. One approach to improve expression would be the introduction of stronger promoter or enhancer elements; but this approach may alter their safety profile by increasing the chance of insertional mutagenesis for NILV that are incorporated into cellular genomes through illegitimate integration [14,15,96,97,98,99,100]. Several studies now show that vectors with less potent enhancer or promoter elements do have an improved safety profile [101,102,103,104,105,106].

A second approach is removing or reducing inhibitors to episomal transgene expression. Bayer *et al.* have shown that removal of *cis*-acting sequences within the U3 region of the vectors LTR improves episomal transgene expression by nearly 3-fold [64]. However, other mechanisms of episomal inhibition may be involved because expression was still below that of the normal, integrating LV control.

A third approach to improved expression is the inhibition of cellular restriction factors. Utilizing the simian immunodeficiency virus auxiliary protein Vpx enabled the inhibition of the myeloid-lineage specific protein SAMHD1. SAMHD1 restricts an early step in the viral life cycle and inhibiting this protein greatly improves the transduction of human and simian myeloid cells [107,108,109,110]. Furthermore, applying this inhibition in conjunction with integrase deficient LV preparations was able to improve episomal expression to levels observed with normal integrating LV [111,112]. Other approaches for improving transgene expression include codon optimization to improve protein production and potency [113,114] and the use of histone deacetylase inhibitors for transgene activation [115].

Combining modifications that increasing transgene expression and reduce integration will be needed to maximize the safety profile of NILV. If expression is low, a higher number of NILV will be required per cell to obtain the therapeutic benefit. The higher numbers of vector episomes will in turn increase the chance of illegitimate integration. For certain gene therapy trials, such as those using *ex vivo* gene transfer of CD34+ hematopoietic cells, the number of cells treated may exceed 5 × 10^8^ cells for an adult. Even with a four log reduction in integration, a significant number of cells will contain integrated proviruses. Therefore efforts to minimize integration and optimizing expression should be considered for both therapeutic and safety reasons.

## 3. Prospects and Applications

Several modifications have been researched to improve the safety and utility of NILV for future clinical applications. These improvements facilitate the development of NILV for the treatment of genetic diseases, infectious diseases, and as important mediators of cell reprogramming. NILV are being developed for clinical applications where integration is not required in order to minimize the risk of genotoxicity by insertional mutagenesis. They also are of interest where transient expression is preferred over sustained gene expression. These applications include vaccinations, cell-type and lineage differentiation, as donor templates for homologous recombination in site-directed integration systems, and as delivery systems for cytotoxic cancer therapies. NILV are also being considered for gene transfer into slowly growing or non-dividing tissues where persistent episomal expression can provide a long-lasting therapeutic effect. An overview of vector components for many of these applications is provided in Table 1.

**Table 1:** Summary of elements included in non-integrating lentiviral vectors (NILV) design. The far right column categorizes the applications depending on the intended purpose. The column of NILV Modifications provides the integrase mutations or Δ*att* (LTR integrase attachment site mutation) used to inhibit integration. Other components of the vector systems are provided in subsequent columns. Abbreviations: iPS, induced pluripotent stem (cell); VSV-G, Vesicular Stomatitis Virus glycoprotein (IND and NJ serotypes if specified); HCV-E1E2-G, Hepatitis C Virus E1E2 glycoproteins; SVGmu, Sindbis virus envelope glycoprotein; ampho MLV, amphotrophic murine leukemia virus; GP64, baculoviral-derived glycoprotein; hAAT, liver specific promoter human α1-antitrypsin; PGK, phosphoglycerate kinase; EF1α, eukaryotic translation elongation factor 1 alpha 1; EFS, short; SV40, simian virus 40 promoter; APOA-II, human liver-specific promoter Apolipoprotein A-II; ET, hepatocyte-specific chimeric promoter; SFFV, Spleen Focus-Forming Virus; CMV, Cytomegalovirus.

	NILV Modification	Disease/Application	Envelope	Promoter	Transgene/Effector	Target	Ref.
Vaccinations	D64V	West Nile Virus	VSV-G	CMV	West Nile Virus Envelope	Dendritic Cells	[93]
	D64V	Malaria	VSV-G IND or NJ & Cocal Virus-G	CMV	*Plasmodium yoelii* Circumsporozoite Protein codon optimized	Dendritic Cells	[116]
	D64E	Hepatitis C Virus	HCV-E1E2-G	CMV	Hepatitis C Virus *NS3* gene	Antigen Presenting Cells	[117]
	D116N	Human Papillomavirus	VSV-G	CMV	Human Papillomavirus 16 E7-Calreticulin fusion	Antigen Presenting Cells	[118]
	D64V	Thymoma	SVGmu	Ubiquitin-C	Ovalbumin, melanoma antigen hgp100 and HIV-1 subtype B *gag*	Dendritic Cells	[119]
	D64V, N120L, W235E & Δ*att*	Hepatitis B Virus	VSV-G	SFFV	Hepatitis B Virus surface antigen	Dendritic Cells	[120]
	D116N	Human Immunodeficiency Virus type 1	VSV-G	CMV	HIV-1 _JR-FL_ gp120 codon optimized	Antigen Presenting Cells	[114]
Cell-Type Differentiation	D64V	Purification of hESC derived progenitors	VSV-G	APOA-II	Green Fluorescence Protein	Hepatic Progenitors	[121]
	D64V	iPS Cell production	VSV-G	EF1α	*OCT4*, *SOX2*, *NANOG*, *LIN28*, *n-Myc* and SV40 Large T Antigen	Fibroblasts	[122]
	D64N & D116N	iPS Cell transgene excision	VSV-G	CMV	Cre recombinase	iPS Cells	[123]
Site-Directed Integration	D64V	Retargetting HIV-1	ampho MLV	SV40	Integrase- *E. coli* LexA repressor fusion protein	*E. coli* LexA recognition sites	[124]
	D64V	Directed Integration	VSV-G	SV40	Integrase-Designed Polydactyl Zinc Finger Protein E2C fusion protein	E2C-recognition sequence	[125]
	D64V	Directed Integration	VSV-G	PGK	Yeast Flpx9 recombinase	Flp-recognition sites	[126]
	D64V	Transposon mediated random integration	VSV-G	CMV, SFFV & SV40	Sleeping Beauty Transposase/Transposase Expression Cassette	Random Integration	[127]
	D64V	Homologous recombination mediated gene modification	VSV-G	N/A	Calmegin targeting cassette	Calmegin (clgn) gene	[128]
	K264E, F185A, D116A, D64A & H12A	Site-directed homologous recombination	VSV-G	CMV	*I-SceI* Nuclease/Homologous recombination repair matrix	*I-SceI* nuclease binding site	[84]
	D64V	Site-specific integration	VSV-G	PGK & SFFV	Zinc Finger Nuclease/ZFN donor template	ZFN-target site at *IL2RG*	[129]
	D64V	“Safe-site”-specific integration	VSV-G	SFFV, PGK & EF1α	Zinc Finger Nuclease/ZFN donor template-GFP expression cassette	*CCR5* and *AAVS1* loci	[130]
	D64V	Site-specific gene modification	VSV-G	EFS	Zinc Finger Nuclease/ZFN donor template	Adenosine Deaminase Locus	[131]
	D64V	Site-specific gene modification	VSV-G	CMV	Transcription Activator-Like Effector Nucleases/TALEN donor template	*COL7A1* gene	[132]
Persistent Episomal Expression	D64V, N120L, W235E, Q148A, K264R & Δ*att*	Stable gene transfer to muscle	VSV-G	SFFV	Green Fluorescence Protein	Muscle Tissue	[61]
	D64E	Stable gene transfer to liver and brain	VSV-G	CMV & hAAT	Green Fluorescence Protein/Luciferase	Brain & Liver Tissue	[64]
	D64V	Stable gene transfer to liver	VSV-G	PGK & ET	Green Fluorescence Protein/Factor IX cDNA	Hepatocytes	[91]
	D64V	Stable gene transfer to liver	VSV-G	ET	Hyperfunctional coagulation factor IX	Hepatocytes	[133]
	D64V	Stable gene transfer to retina and brain	VSV-G	CMV & SFFV	Green Fluorescence Protein	Ocular & Brain Tissue	[89]
	D64E	Stable gene transfer to brain	VSV-G	CMV	Green Fluorescence Protein/Luciferase	Brain Tissue	[58]
	N region RRK motif to AAH	Stable gene transfer to brain	VSV-G	CMV	Green Fluorescence Protein	Neural cells	[62]
	D64V	Stable gene transfer to central nervous system	VSV-G, GP64 & Rabies-G	SFFV	Green Fluorescence Protein	Brain and Spinal Cord	[63]
	D64V	Stable gene transfer to spinal cord	VSV-G & Rabies-G	CMV	Green Fluorescence Protein	Spinal Cord	[134]

Vaccination is an application where only transient expression is required and NILVs have been shown to stimulate an efficient and sustained immune response [112,119,135,136]. Preclinical studies of NILVs have demonstrated immune responses against human papillomavirus (HPV), malaria, HIV-1 and the hepatitis B and C viruses [93,114,116,117,118,120] thus showing their potential for use in vaccine development.

One application where transient expression is preferred over sustained expression is in cell reprogramming. This includes creation of induced pluripotent stem (iPS) cells and differentiation of iPS or embryonic stem (ES) cells into a lineage of interest. While somatic cells have been successfully reprogrammed into iPS cells using integrating vectors [137], the factors for inducing pluripotency are not necessary beyond initial reprogramming and constitutive expression of the factors has been shown to be harmful [137,138,139,140]. Continued expression can be oncogenic [140] and can also affect differentiation of iPS cells into other lineages [141]. Transgene free iPS cells have been produced using integrating LV followed by excision with NILVs after reprogramming [123] as well as by transient expression using both non-integrating adenoviral vectors [142,143] and NILV [122]. NILV have also been successful in differentiating ES cells into specific progenitors [121].

Another promising application for NILV is their use as templates for site-directed integration systems. A variety of systems are available that can direct integration to genomic “safe loci” or by altering the integration pattern to avoid transcriptional units with the hope of minimizing gene dysregulation. Integration can be directed to sequence-specific motifs in less intragenic regions by combining NILVs with an integrase protein fused to a DNA-binding protein such as the *E. coli* LexA repressor [124,144,145] or a synthetic polydactyl zinc finger protein E2C [125]. Another approach is combining recombinases or transposases transiently with NILV to facilitate integration at specific sites [126,127,146]. Third, NILVs can be designed to promote site specific homologous recombination (HR) [128]. Taking this a step farther, others have combined NILV with a rare cutting nuclease for targeted recombination at specific sites by HR [84]. Still others have used NILV as templates for HR along with engineered zinc finger nucleases (ZFNs) or transcription activator-like effector nucleases (TALENs) [129,130,131,132]. Increasing clinical utility is expected as these systems are optimized to reduce off-target integrations and increase the efficiency of delivery. 

NILV have potential utility in cytotoxic cancer therapies. One approach takes advantage of abnormal expression levels of miRNA which are found in many tumor types [147]. *In vitro* and *in vivo* studies have shown miRNAs to have antitumorigenic properties [148]. Recent studies have shown the utility of non-integrating adeno-associated virus in suppressing tumor growth in lung [149,150] and liver cancers [151,152]. Non-integrating vectors offer the advantage of minimizing effects from transgene expression in normal cells. This targeting of miRNA provides the backdrop for developing NILV for similar applications.

While there are many applications for transient expression of NILV, there is also great promise in their utilization for persistent episomal expression in non-proliferating post-mitotic cells. In this regard, NILV have been found to successfully provide long-lasting *in vivo* expression in several organs. Injections of integrase deficient LV and att site mutant NILV into mouse muscle were found to provide levels of transgene expression similar to wild-type LV for up three months post-transduction [61]. NILV have also been used to transduce the liver resulting in stable transgene expression for up to six months [64] and could provide therapeutic levels of transgene expression [91,133]. The retina transduced with NILVs has been shown to provide transgene expression for up to nine months in mice [89]. NILV were used to successfully transduce the brain and spinal cord allowing for efficient transgene expression from 2 weeks up to 4 months post-transduction [58,62,63,89,134].

## 4. Conclusions

A growing number of studies are demonstrating the potential utility of NILV in human gene therapy. Point mutations in integrase or the LTR att sites greatly reduce integration, and additional modifications to the vector or the target cell can further decrease illegitimate integration. Importantly, there have been a number of key studies that have optimized the expression of NILV. While further improvements are being evaluated, the current tools available are suitable for clinical use, and human trials could be conducted in the near future.
